# The Effects and Mechanisms of Low-Intensity Pulsed Ultrasound on Bone Remodeling: From Laboratory to Clinic

**DOI:** 10.3390/biom15101351

**Published:** 2025-09-23

**Authors:** Bo Zong, Weikang Sun, Chao Cai, Peng Shang

**Affiliations:** 1 School of Life Sciences, Northwestern Polytechnical University, Xi’an 710072, China; zongbo@mail.nwpu.edu.cn (B.Z.); sunwk@mail.nwpu.edu.cn (W.S.); caichao@mail.nwpu.edu.cn (C.C.); 2 Key Laboratory for Space Bioscience and Biotechnology, Institute of Special Environmental Biophysics, School of Life Sciences, Northwestern Polytechnical University, Xi’an 710072, China; 3 Research & Development Institute, Northwestern Polytechnical University, Shenzhen 518057, China

**Keywords:** low-intensity pulsed ultrasound, fracture, osteoporosis, piezoelectric effect, mechanical effect

## Abstract

Decades of research and applications have demonstrated that low-intensity pulsed ultrasound (LIPUS) has a certain therapeutic effect on diseases involving bone remodeling. LIPUS operates in a pulsed-wave mode at low intensity, ensuring efficient transmission of acoustic energy to target tissues, thereby providing non-invasive physical stimulation for therapeutic purposes. Bone remodeling refers to the dynamic renewal process of bone tissue that is jointly completed by multiple cells in the bone metabolic microenvironment. LIPUS influences the basic biological processes of bone remodeling in the skeletal system through mechanical, piezoelectric, and thermal effects on bone tissue, triggering a series of biochemical reactions. This article begins with the discovery of ultrasound and research on bone remodeling, introduces the basic parameters and application devices of LIPUS, and reviews the clinical applications of and basic research on LIPUS in bone remodeling disorders. Focusing on the intersection and integration of biomedical fundamentals and ultrasound science, it analyzes the biological and physical mechanisms of LIPUS in research on and applications of bone remodeling disorders and investigates the basic research questions and clinical transformation application scenarios in this field.

## 1. Introduction

Bones are an integral part of the vertebrate body and perform several important functions, such as facilitation of movement, support, protection of soft tissues, storage of bone marrow, and storage of calcium and phosphate [1,2]. Bone tissue is a complex composition consisting of two interrelated components. The first component is connective tissue, comprising an extracellular matrix; organic collagen fibers (which account for approximately 90% of matrix proteins); proteins such as osteocalcin, osteopontin, fibronectin, and thrombospondin 2; inorganic hydroxyapatite; trace amounts of potassium, magnesium, sodium, strontium, and calcium salts; and water [3,4,5,6]. The other component is the cellular component, mainly including bone marrow mesenchymal stem cells, osteoblasts, osteocytes, and osteoclasts [6,7,8]. Bone undergoes continuous renewal through the process of bone remodeling. This process, which is fundamentally a dynamic balance between bone resorption and formation, is orchestrated within specialized bone multicellular units (BMUs). Within these units, intercellular communication between osteoclasts (and their precursor cells) and cells of the osteoblast lineage (osteoblasts, osteocytes, and bone-lining cells) ensures this precise equilibrium [9,10]. During bone remodeling, osteoclasts degrade the organic matrix components and acidify the extracellular environment by releasing enzymes including tartrate-resistant acid phosphatase (TRAP) and hydrogen ions (H^+^), thereby facilitating the dissolution of hydroxyapatite crystals. Simultaneously, osteoblasts are responsible for secreting proteins to form the organic matrix and guiding the mineralization of the newly formed osteoid. Mature osteoblasts may subsequently differentiate into bone-lining cells or osteocytes, or undergo apoptosis. It is important to emphasize that osteoclasts and osteoblasts do not operate in a sequential replacement manner; rather, they function concurrently in a spatially coordinated manner within the BMU.

Bone remodeling is the core biological process for maintaining bone homeostasis [11]. However, various external physical, chemical, and biological factors can disrupt bone remodeling, leading to the occurrence of numerous bone remodeling disorders. Common bone remodeling disorders include fractures, osteoporosis, and nonunion. Fractures are characterized by the complete or partial disruption of bone continuity, resulting in significant changes in the mechanical and biochemical microenvironment of bone cells [12]. Nonunion is a complication of fracture healing in which there is incomplete healing within 9 months after injury or no signs of bone callus formation on X-ray films within the subsequent 3 months [13]. Osteoporosis is a systemic metabolic bone disease characterized by bone mass reduction and destruction of the bone tissue microstructure, increasing bone fragility and the risk of fractures [14].

Currently, the treatment methods for bone remodeling disorders include surgical treatment, drug treatment, and physical therapy. Fractures and nonunion are mainly treated by surgical and physical methods, such as plaster fixation, joint replacement, electromagnetic fields, shock waves, and LIPUS treatment. Osteoporosis is mainly treated with drugs, supplemented by lifestyle interventions such as weight-bearing exercise. Anti-osteoporosis drugs mainly include vitamin D, calcium supplements, bisphosphonates, selective estrogen receptor modulators, calcitonin, denosumab, and teriparatide [15]. Although drug treatment is effective, it has problems such as long treatment cycles, poor patient compliance, high costs, and adverse drug reactions [16]. Therefore, LIPUS, as a non-invasive physical treatment method, has attracted increasing attention for its effectiveness, safety, simplicity, and cost-effectiveness in treating bone remodeling-related diseases. Furthermore, the efficacy of LIPUS is supported by a growing body of research elucidating its physical and biological mechanisms of action on bone remodeling.

Clinical studies have shown that LIPUS with specific parameters has preventive and therapeutic effects on bone remodeling disorders, promotes the healing of fractures and nonunion [17], and has a certain therapeutic potential for osteoporosis, which has also been observed in animal models [18]. In addition, cell experiment studies have shown that LIPUS with specific parameters promotes the differentiation of bone marrow mesenchymal stem cells and osteoblasts, promotes the proliferation of osteoblasts, inhibits osteoclastogenesis, and alters a variety of bone cell–cell communication processes, thereby influencing bone remodeling [19,20,21].

This article focuses on research on and applications of LIPUS in bone remodeling disorders. This review provides a comprehensive examination of LIPUS and its role in the management of bone remodeling disorders. Section 2 traces the historical discovery of therapeutic ultrasound and key milestones in the development of LIPUS. Section 3 details the classification of ultrasound modalities and presents a critical analysis of its fundamental parameters, including intensity, frequency, pulse repetition frequency, pulse width, duty cycle, and exposure time. Section 4 surveys currently available LIPUS devices for both research and clinical applications. Section 5 evaluates clinical evidence and ongoing controversies regarding the efficacy of LIPUS in treating fractures, nonunions, and osteoporosis. Section 6 reviews findings from animal studies and discusses cellular-level mechanisms involving osteoblasts, osteoclasts, mesenchymal stem cells, chondrocytes, and osteocytes. Section 7 elaborates on the biophysical mechanisms of LIPUS, covering mechanical effects—such as acoustic radiation force, acoustic streaming, and cavitation—piezoelectric effects, thermal effects, and their interactions with biological tissues. Finally, Section 8 summarizes current challenges and future research directions related to mechanistic understanding, technological innovation, and clinical translation of LIPUS.

## 2. Discovery and Development of Ultrasound in Bone Remodeling Disorders

**Figure 1:**
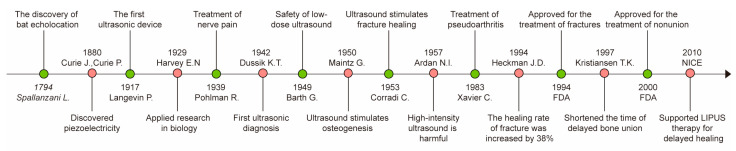
The discovery of LIPUS as physical therapy in bone remodeling disorders [22,23,24,25,26,27,28,29,30,31,32,33,34,35,36,37].

From the discovery of ultrasound to the development of the first ultrasound device based on the piezoelectric effect, and further to its gradual application in biomedical physical therapy and diagnosis, the developmental process is illustrated in Figure 1 [22,23,24,25,26,27].

However, due to the high intensity of the ultrasound used in early studies, people argued that ultrasound treatment might cause bone damage. It was not until 1949 that Barth discovered that low-dose ultrasound had a safe and controllable biological effect on bones and surrounding tissues and would not cause pathological damage [28]. In 1950, Maintz published the first report describing the use of ultrasound to stimulate bone formation: although high-intensity ultrasound of 2500 mW/cm^2^ caused thermal damage and reduced bone callus formation, lower-dose ultrasound of 500 mW/cm^2^ led to the formation of a new periosteum after rabbit osteotomy [29]. In 1953, Corradi discovered that using ultrasound stimulation could accelerate the healing of rabbit radius fractures [30]. In 1957, Ardan from the Mayo Clinic reported that high-intensity ultrasound (5000–25,000 mW/cm^2^) arrested the healing of dog femurs, leading to the formation of dense fibrous tissue and necrosis [31]. In 1964, to reduce the adverse effects of high-intensity ultrasound on bone tissue, Shiro began to treat the tibia of rabbits with 200 mW/cm^2^ LIPUS and confirmed that it could accelerate tibia growth [32]. Subsequently, more researchers began to reduce the signal intensity output of ultrasonic transducers and use them in clinical research. In a clinical study beginning in 1979, Xavier and Duarte were the first to report that non-invasive ultrasound could treat patients with pseudoarthritis and delayed fracture healing, and that the cure rate of pseudoarthritis was 70% [33]. In 1994, Heckman et al. reported that 30 mW/cm^2^ LIPUS treatment could reduce the healing time of human tibial fractures by 38% [34]. Based on this and other early clinical evidence, the US Food and Drug Administration (FDA) approved the application of LIPUS for the treatment of fresh fractures in October 1994, which was an important milestone in the clinical application of LIPUS in orthopedics [35]. Kristiansen et al. reported in 1997 that LIPUS treatment shortened the duration of delayed bone healing, accelerated the radiographic healing process, and significantly reduced the loss of reduction [36]. Following this evidence, the US FDA approved LIPUS for the treatment of nonunion in February 2000 [35]. Since 1994, from the clinical treatment of bone remodeling disorders to laboratory molecular cytology experimental research, new basic research and clinical application achievements related to LIPUS in bone remodeling-related diseases have continuously emerged. It has been repeatedly discovered and proven that with certain parameters and characteristics, LIPUS can promote bone formation and inhibit bone resorption. In 2010, the National Institute for Health and Care Excellence (NICE) in the UK issued a statement supporting the use of LIPUS to shorten the fracture healing time and provide clinical benefits, especially in cases of delayed healing [37]. In 2017, Poolman published a clinical practice guideline that further summarized and standardized the application of LIPUS in fracture healing [38]. These studies have opened a new door for the application of ultrasound in the treatment of bone remodeling disorders (Figure 1). In recent years, LIPUS has been the focus of numerous studies, investigating its ability to stimulate bone formation in fractures and nonunions [39].

## 3. Classification and Parameter Studies of Ultrasound

### 3.1. Classification of Ultrasound

Ultrasound is a short-wavelength sound wave with good directivity, strong penetrability, and concentrated energy above 20 kHz [40]. Pulsed ultrasound is a specific type of ultrasound output in the form of periodic pulse waves [41]. According to the exposure intensity, pulsed ultrasound can be divided into LIPUS (<3 W/cm^2^) and high-intensity pulsed ultrasound (≥3 W/cm^2^). According to the dose, ultrasound can be further divided into three groups: low dose (<1 W/cm^2^), medium dose (1–2 W/cm^2^), and high dose (2–3 W/cm^2^) [42]. According to the frequency, ultrasound can be divided into low-frequency ultrasound (20–200 kHz) for industrial and therapeutic applications, medium-frequency ultrasound (0.7–3.0 MHz) in therapeutic medicine, and high-frequency ultrasound (1–20 MHz) for medical diagnosis [43].

### 3.2. Research on Ultrasound Parameters

Important parameters of LIPUS include intensity, frequency, pulse repetition frequency, pulse duty cycle, pulse width, and exposure time. The vast majority of published studies on LIPUS for bone remodeling have employed devices similar to the commercial Exogen system (SAFHS, Exogen, Piscataway, NJ, USA) [44], which employs parameters as summarized in Table 1.

**Figure 2:**
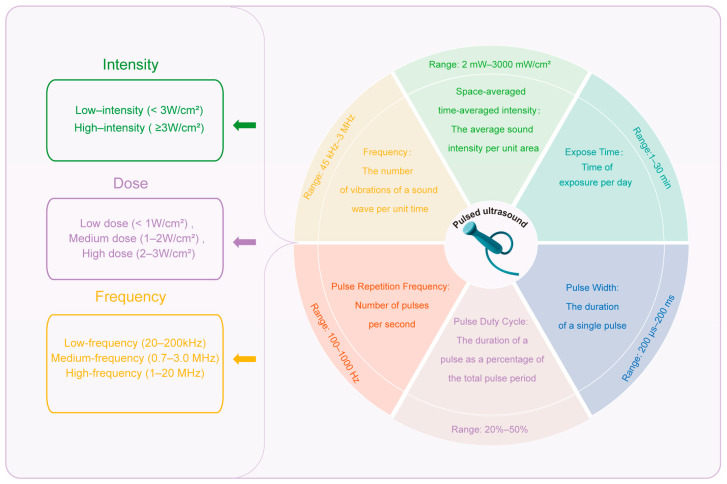
Classification of pulsed ultrasound and definitions/ranges of key parameters. The right panel presents the definitions and typical ranges of six specific ultrasound parameters, whereas the left panel illustrates three established classification approaches for pulsed ultrasound modalities.

Currently, the LIPUS devices that are commonly used in the research on bone remodeling disorders usually operate in the frequency range of 45 kHz–3 MHz, with a pulse repetition frequency in the range of 100–1000 Hz, a pulse width of 200 μs–200 ms, a pulse duty cycle of 20–50%, an intensity between 2 and 3000 mW/cm^2^, and a daily exposure time of 1–30 min [45,46,47,48,49,50,51]. We show the classification, parameter definitions, and usage ranges of pulsed ultrasound (Figure 2). How these ultrasonic device parameters can be adjusted to enhance their therapeutic effect on bone remodeling diseases remains to be discussed. We need to understand the meaning and role of each ultrasonic parameter, which are elaborated below.

#### 3.2.1. Intensity

An extension of a guideline for reporting ultrasound exposure conditions emphasizes that acoustic intensity must be reported in research studies [52]. Acoustic intensity is a measure of the energy flow per unit time per unit area within an acoustic medium, encompassing several types such as spatial-peak pulse-average intensity (I_sppa_), spatial-peak temporal-average intensity (I_spta_), and spatial-average temporal-average intensity (I_sata_) [45,53]. Understanding the definitions and calculation methods of different intensity parameters is crucial for enabling cross-study comparisons and ensuring reproducibility. We have elaborated on these three types of ultrasound intensities in Table 2. The calculations of I_sppa_ and I_spta_ are primarily applied in research on transcranial ultrasound stimulation [53]. In contrast, Isata is extensively utilized and reported in studies investigating the effects of LIPUS on bone [45].

Existing studies have shown that different levels of I_sata_ can induce distinct biological effects. For example, pulsed ultrasound at 2.2 W/cm^2^ (applied for 20 min/day over 6 weeks) induced pathological changes in growing bones, whereas no adverse effects were observed at 0.5 W/cm^2^ under identical conditions [54]. Similarly, some studies have indicated that a pulsed ultrasound intensity of 150 mW/cm^2^ leads to higher RANKL gene expression in cementoblasts 24 h after a 15 min exposure compared to 30 mW/cm^2^ [55]. Furthermore, Sun et al. demonstrated that among LIPUS intensities ranging from 15 to 150 mW/cm^2^, a dose of 150 mW/cm^2^ most effectively alleviated bone loss and osteoporosis in ovariectomized (OVX) rats [56]. Supporting these findings, another study showed positive correlations between LIPUS intensity and both bone volume fraction and trabecular thickness in OVX rats. Specifically, compared to the OVX control group, the 100 mW/cm^2^ group exhibited a 33% higher bone volume and a 23% greater volume than the 5 mW/cm^2^ treatment group. Significant increases in trabecular thickness were also observed at 30 mW/cm^2^ and 100 mW/cm^2^ relative to OVX controls, suggesting intensity-dependent roles in influencing bone formation [18]. However, it is important to note that excessively high ultrasound intensities (>1.0 W/cm^2^) can cause adverse effects including premature epiphyseal growth plate closure, slippage, displacement, bone sclerosis, diaphyseal fractures, fibrosis, and delayed fracture healing [30,31,57]. In contrast, LIPUS at intensities below 0.1 W/cm^2^ shows no known adverse effects on bone tissue [35,58,59]. Collectively, these findings underscore the significant role of ultrasonic intensity in bone remodeling and highlight the need for further research to optimize intensity parameters for different bone disorders.

#### 3.2.2. Frequency

Frequency refers to the number of sound wave vibrations per unit time, yet its role in therapeutic outcomes remains underexplored [60]. Notably, ultrasound penetration depth is inversely proportional to frequency [61,62,63]. For example, at 1 MHz, penetration depth measures 7 mm in bone, 30 mm in muscle, and 37 mm in skin and subcutaneous tissue [64]. Furthermore, higher frequencies improve resolution but reduce penetration, whereas lower frequencies enhance penetration at the expense of resolution [65]. Studies also indicate a positive correlation between frequency and cavitation density [66], suggesting that frequency selection should be optimized for specific tissue depths and treatment objectives. Crucially, frequency-specific responses are evident in bone cells. For instance, modulated-frequency ultrasound (with 1–2 MHz variation) enhanced bone formation and bone quality in osteoporotic mice compared to fixed-frequency LIPUS [60]. Similarly, low frequencies (e.g., 45 kHz) promoted the production of PGE2 in human osteoblasts, and PGE2 in turn enhanced osteoclast formation [48]; amplitude-modulated signals at 45/100 kHz enhanced osteoblast mineralization more effectively than 1 MHz LIPUS [67]. Moreover, stimulation at 1–1.5 MHz improved osteoblast proliferation and early differentiation [68,69], while 2 MHz preferentially induced osteogenic differentiation in stem cells [70]. Collectively, these findings highlight the potential of frequency modulation to optimize energy delivery and elicit targeted cellular responses.

#### 3.2.3. Pulse Repetition Frequency

Pulse repetition frequency (PRF) can differentially modulate cellular behavior, as evidenced by divergent responses influenced by ultrasound parameters and cell types. Samuel et al. observed that 1 kHz PRF induced minimal cell mortality and damage in microbubble-bound macrophages compared to lower PRFs (0.1–100 Hz) [71]. In contrast, Monici et al. reported that 1 Hz PRF enhanced proliferation of osteoclast precursors, while higher PRFs (100–1000 Hz) suppressed it [72].

#### 3.2.4. Pulse Width

Pulse width (μs) determines stimulation mode: longer pulses sustain mechanical effects, while shorter pulses use repetition for cumulative impact. High intensity with long pulses increases thermal risk, reduced by shorter pulses [73]. Bioeffects stem from both thermal and mechanical mechanisms—enhanced by longer pulses and minimized by shorter ones [74,75]. Samuel et al. reported that increasing the pulse width (0–100 ms) at a pulse repetition frequency (PRF) of 1000 Hz elevated macrophage mortality, which demonstrated cellular damage dependent on pulse width [76]. Although a pulse width of 200 μs is widely used in LIPUS to enhance osteoblast activity and osteogenesis, a systematic review by Luiz et al. suggested that longer pulses might improve bone mineral density recovery—though this conclusion requires further validation [77]. Collectively, these findings demonstrate the need for further investigation into the cellular effects of different pulse widths and for mechanistic studies to clarify the underlying response pathways.

#### 3.2.5. Pulse Duty Cycle

The pulse duty cycle is the percentage of time a pulse is active within a single period. Zhou et al. observed that a high duty cycle (10%) exhibited a greater capacity to dissolve platelet-rich thrombi compared to a low duty cycle (0.03%) [78]. In skeletal system applications of LIPUS, a 20% duty cycle is most frequently employed. Nevertheless, there is presently limited comparative research on the effects of different duty cycles.

#### 3.2.6. Exposure Time

Exposure time refers to the duration of daily exposure. In male Wistar rats with post-traumatic joint contracture (PTJC), treatment with LIPUS at 120 mW/cm^2^ for 20 min significantly reduced intra-articular adhesions compared to a regimen of 30 mW/cm^2^ for 5 min. The combined effects of intensity and duration were additive rather than synergistic, which highlighted the importance of tailoring parameters to therapeutic objectives and specific disease models [79]. Additionally, Warden et al. reported that in a femoral fracture model using adult male rats, daily LIPUS exposure at 100 mW/cm^2^ for 20 min did not improve fracture healing by day 25 but led to significant enhancements in bone mineral content, bone size, strength, and stiffness by day 40 compared to controls [80]. In cellular studies, Monici et al. observed that in the FLG 29.1 osteoclast precursor line, 15 min of LIPUS exposure increased proliferation at both 24 and 48 h, whereas 60 min of treatment resulted only in a mild increase at 24 h followed by a pronounced decline at 48 h [72]. Similarly, Zhou et al. demonstrated that in the J774A.1 macrophage line, LIPUS exposure induced strong actin polymerization in a time-dependent manner, which peaked at 30 min after stimulation [81]. These studies indicate that both daily exposure duration and total treatment days influence the cumulative exposure dose, resulting in distinct biological effects—particularly as treatment efficacy varies across different stages of fracture healing. Therefore, precise control of exposure time and dose is critical for minimizing discrepancies in experimental design and enabling standardized comparisons.

In fundamental research and clinical applications of ultrasound, the precise conceptualization of “acoustic dose” is crucial. Building on concepts from radiation therapy, Nandi et al. proposed a key theoretical framework for ultrasound dose. This framework breaks down dose into four levels: absorbed dose (energy absorbed by tissues, related to thermal and mechanical effects), equivalent dose (incorporating parameters such as frequency to characterize biophysical mechanisms), effective dose (accounting for tissue-specific responses), and cumulative dose (reflecting the additive effects of exposure history). Dose is tentatively defined as the integral of amplitude over time (i.e., intensity-time integral), which is a practical and mechanism-neutral parameter relevant to various potential biophysical effects (thermal, mechanical, cavitation). This framework provides a standardized foundation for current research and anticipates future refinements through weighting optimizations based on mechanisms and target characteristics (e.g., frequency, ion channel density), thereby guiding more precise fundamental and applied studies on the biological effects of ultrasound [82].

## 4. LIPUS Devices for Bone Remodeling Disorders

**Figure 3:**
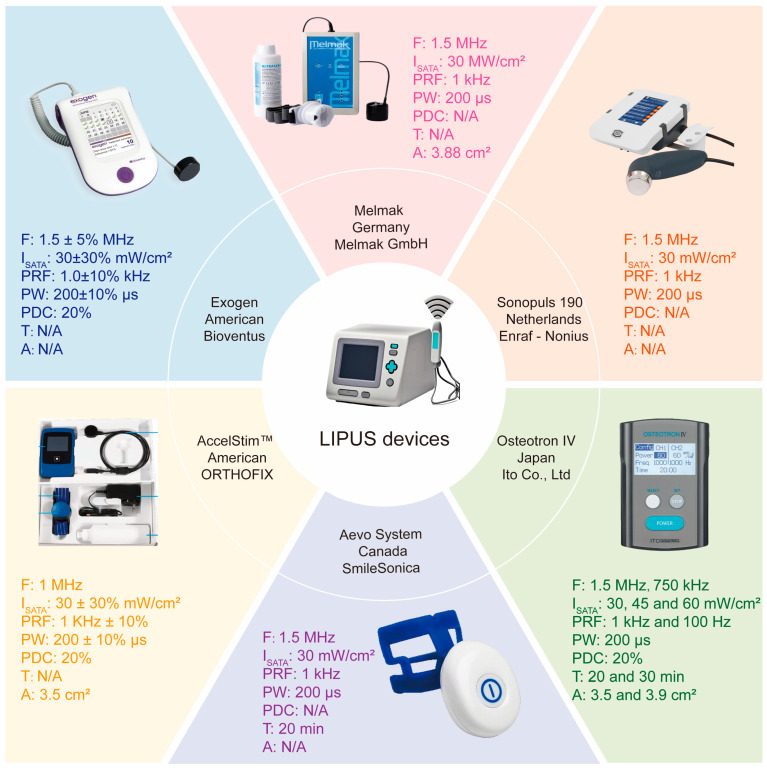
LIPUS devices and their parameters for experimental research and clinical use in different countries. Six LIPUS devices from five countries worldwide are listed, including the device names, the companies they belong to, and their countries of origin. Moreover, the different parameters that each device can modulate are listed in detail. LIPUS, low-intensity pulsed ultrasound; F, frequency; I_SATA_, space-averaged time-averaged intensity; PRF, pulse repetition frequency; PW, pulse width; PDC, pulse duty cycle; T, exposure time; A, area of ultrasonic transducer; N/A, not available or not applicable.

Since LIPUS therapy received FDA approval in 1994, numerous LIPUS medical devices for bone remodeling disorders have emerged globally [83]. Classified as non-invasive bone growth stimulators (BGSs) by the FDA, these devices use transducers to deliver mechanical waves non-invasively to fracture sites, thus promoting healing [84]. Focusing on devices for bone remodeling disorders, LIPUS devices are portable due to their low intensity compared with other clinical ultrasound tools. The first commercial LIPUS product was the EXOGEN^®^ device, and commonly used skeletal growth stimulators include Bioventus Exogen 4000+™, Exogen 3000™, Exogen 2000+™, and Exogen 2000™ [85]. Through a survey of frequently used ultrasound devices in the scientific literature on LIPUS for bone remodeling disorders, we summarize several LIPUS instruments used to treat bone-related disorders in different countries and list their relevant parameter information (Figure 3).

As shown, most commercial devices use low-intensity, medium-frequency, and low-dose pulsed ultrasound. Current experimental and clinical studies typically employ the following parameters: 30 mW/cm^2^ intensity, 1.5 MHz frequency, 20% pulse duty cycle, 1 kHz PRF, 200 μs pulse width, and 20 min of daily exposure [86]. This leads us to pose several key questions: What is the clinical efficacy of LIPUS in treating different bone remodeling disorders? What are the biological and physical mechanisms underlying LIPUS action in bone remodeling? To address these questions, we conducted a literature retrieval and summarized our findings in the subsequent sections.

## 5. Clinical Applications of LIPUS in Bone Remodeling Disorders

LIPUS has primarily been studied for treating bone remodeling disorders, with the majority of research focusing on fractures, although it also demonstrates therapeutic effects on nonunion and delayed healing. Clinical studies on osteoporosis are still in their infancy with inconsistent results, but the current research suggests that LIPUS holds potential for treating this condition. Clinically, LIPUS is applied using ultrasonic transducers on fracture sites, osteoporotic regions, or inflamed areas that are coated with medical ultrasound coupling gel, evaluating its effects on tissue healing, bone mass recovery, swelling, and pain to investigate its impact on bone remodeling disorders. Table 3 summarizes clinical studies using LIPUS for bone remodeling disorders.

### 5.1. Fractures and Nonunion

Numerous studies have shown that LIPUS effectively promotes callus formation at fracture sites, shortens the healing time, enhances bone regeneration and growth, and reduces pain [87,88,89,90]. A meta-analysis by Lou et al. confirmed the positive effects of LIPUS on fresh fractures in adults [104]. A cohort study involving 4190 fresh fracture patients aged 30–79 found that the healing rate in the LIPUS-treated group was 96%, significantly higher than the expected average of 93%, with a notably shorter treatment time, indicating that early LIPUS intervention benefits fracture healing [105]. However, some reports noted no statistically significant effect of LIPUS on lateral malleolus fracture healing [91]. The negative findings may be attributed to the fact that fracture healing generally occurs within six weeks, and clinical measurements in this study began at the 6-week time point, potentially leaving no window to detect measurable differences. Additionally, radiographic fracture line visibility did not fully correlate with clinical healing, and a slight trend toward increased callus formation in the LIPUS group was not statistically significant due to the limited sample size. Major complications associated with fracture repair include osteomyelitis, nonunion, and delayed healing [106]. Although LIPUS cannot replace surgical debridement, it may hold therapeutic potential against osteomyelitis through anti-inflammatory mechanisms, such as promoting M2 macrophage polarization [107,108]. However, owing to the limited number of experimental studies on LIPUS for osteomyelitis—coupled with clinical limitations including a shortage of large-scale trials, restricted efficacy in deep-seated infections, and multifaceted factors affecting treatment outcomes—LIPUS may currently be regarded merely as a promising adjunctive approach rather than a standalone therapeutic option.

LIPUS is also used to treat nonunion and delayed healing after osteotomy, accelerating distraction osteogenesis and callus maturation [92,93,94,95,96,97,98,99]. LIPUS treatment accelerated the clinical healing of delayed fibular union by increasing osteoid thickness, mineral deposition rate, and bone volume in nascent bone formation areas, but did not alter osteoid thickness or mineral apposition rate in cancellous bone regions, primarily due to its target specificity for early-stage osteoblasts, limited energy penetration into mature bone structures, and minimal influence on differentiated or functionally stable osteoblasts in cancellous bone [92]. The other one study reported that LIPUS stimulation had no effect on the bone healing rate in distraction osteogenesis, possibly due to smoking—a known inhibitor of bone healing—among participants, highlighting its impact on managing such patients [109]. Similarly, a double-blind, randomized, placebo-controlled trial found LIPUS ineffective for lower limb stress injuries, which might be attributed to deviations in LIPUS treatment parameters (117 mW/cm^2^, 200 ms) from established standards, a limited sample size predominantly of elderly females, unmonitored treatment compliance due to home-based device use, unrecorded activity levels, and weight-bearing status during the study, as well as subjective differences in pain tolerance among participants [100]. Notably, a study on Chevron osteotomy subjects showed radiological evidence of enhanced bone formation with LIPUS but no clinical improvements at the 1-year follow-up [101]. This discrepancy may be explained by several factors: the restricted follow-up intervals (only 6 weeks and 12 months), which may have missed transient therapeutic effects during early healing; the high innate healing capacity of participants with normal bone stock, potentially masking any incremental benefit from LIPUS; and the absence of a clear relationship between radiographic enhancement and clinical functional outcomes, indicating that improved bone formation did not lead to symptomatic or functional gains within one year.

These clinical trials confirm that LIPUS treatment promotes healing in fractures and nonunion, although inconsistent results exist for specific fracture sites, likely due to variations in ultrasound devices, treatment parameters, sample size, follow-up duration, patient compliance, and fracture location, and other factors. As shown in Table 3 and supported by the studies mentioned above, the LIPUS parameters commonly used for nonunion treatment include an intensity of 30 mW/cm^2^, frequency of 1.5 MHz, duty cycle of 20%, pulse repetition frequency of 1 kHz, pulse width of 200 μs, and a daily exposure duration of 20 min. While current clinical evidence supports the efficacy of these standardized parameters, we acknowledge that exploring optimized parameter combinations tailored to nonunions represents an important direction for future research. For instance, studies using alternative parameters (e.g., 1.0 MHz, 500 mW/cm^2^, 10% duty cycle) have also demonstrated improved outcomes in rib fracture healing [87]. It is only through comparative clinical studies involving diverse LIPUS parameters that the most effective settings can be identified. Given the limited global clinical research on LIPUS for fractures and nonunion, future studies require rigorous experimental designs and standardized parameter control.

### 5.2. Osteoporosis

While LIPUS stimulation accelerates bone formation in fractures and nonunion, clinical reports on osteoporosis are limited and controversial. Ali et al. suggested that numerous in vivo animal and clinical trials have shown that LIPUS stimulation not only prevents bone loss but also restores bone mass, and that its systemic application could offer significant clinical benefits for treating osteoporosis [110]. Leung et al. exposed periosteal cells derived from human tibiae to LIPUS and observed dose-dependent enhancements in cell proliferation, alkaline phosphatase (ALP) activity, osteocalcin (OCN) and vascular endothelial growth factor (VEGF) secretion, calcium nodule formation, and osteoblastic differentiation [111]. An exploratory clinical trial found that LIPUS stimulation for 3 months after concentrated autologous bone marrow aspirate transplantation accelerated osteogenesis and angiogenesis in avascular necrosis of the femoral head [112]. However, LIPUS was found to have no significant effect in preventing postmenopausal distal radius bone loss in older Chinese women, with the researchers suggesting that substantial attenuation of ultrasound transmission by the selected bone site, reduced or absent responsiveness of bone to mechanical stimuli in late adulthood of the subjects, and limitations in sample size and follow-up duration may have led to the underestimation of the potential local osteogenic effects of LIPUS treatment [103]. Negative findings in spinal cord injury-induced osteoporosis suggested that ultrasonic acoustic properties, such as poor cortical bone penetration, limit its efficacy [113]. Notably, while LIPUS has shown promise in treating fractures and nonunion, robust clinical data for its use in treating primary osteoporosis continues to be scarce. The transition from in vitro and animal studies to large-scale clinical trials is essential, along with addressing heterogeneity in ultrasound parameters, disease models, exposure durations (3 weeks to 18 months), treatment sites, and participant demographics that contribute to conflicting results. Standardized protocols for subject selection, device parameters, exposure methods, and follow-up periods are critical to generating reliable clinical evidence.

## 6. Laboratory Studies on LIPUS in Bone Remodeling Disorders

### 6.1. Animal Studies

In recent years, experimental studies on LIPUS in animal models of skeletal diseases have gradually emerged. Increasing evidence confirms that with specific parameters, LIPUS can promote fracture healing, treat nonunion, enhance callus formation and angiogenesis in osteoporotic fractures, stimulate bone formation in osteoporotic animal models, improve bone defect healing rates, optimize bone microstructure, and increase BMD. However, some studies have found that LIPUS has no significant effect on bone healing and formation. Both positive and negative results from relevant animal studies are summarized in Table 4.

#### 6.1.1. Fracture and Nonunion Models

In recent years, an increasing number of laboratory studies have aimed to investigate the role of LIPUS in fractures and nonunions, utilizing common animal models including dogs, rabbits, rats, guinea pigs, sheep, and horses. Early animal research revealed that high-intensity ultrasound stimulation (5000–25,000 mW/cm^2^) caused tissue necrosis and halted bone healing in animals [127], prompting subsequent experiments to adopt ultrasound intensities below 3000 mW/cm^2^ across different models. Maintz used low-intensity ultrasound (500–2000 mW/cm^2^) to treat rabbit limbs and observed formation of new periosteal bone [29], while Corradi first successfully induced new callus at fracture sites using 1500 mW/cm^2^ continuous ultrasound [128]. Murolo demonstrated that ultrasound stimulation accelerated ulnar fracture healing in guinea pigs [129], and Shiro achieved tibial growth in rabbits with 200 mW/cm^2^ ultrasound signals [32]. Klug et al. reported that LIPUS exposure accelerated tibial fracture healing in a rabbit model of closed leg fractures and secondary fracture healing [114], while Heybeli et al. confirmed increased bone density and radiographic fracture healing in rat femurs using LIPUS [115]. Shimazaki et al. found that LIPUS application enhanced callus maturation compared to controls, as validated through biomechanical, densitometric, and radiographic assessments [116]. Furthermore, a sheep osteotomy model demonstrated that LIPUS stimulation significantly accelerated fracture healing, increased cortical BMD, and improved the lateral bending strength of healed fractures [118]. In OVX rats, LIPUS stimulation enhanced callus formation, angiogenesis, and callus remodeling during osteoporotic fracture healing [121]. In aged mice, LIPUS halved the endochondral bone remodeling period to 10 days, accelerating femoral fracture healing [122]. Additionally, a review summarized LIPUS’s effects on tendon–bone healing after anterior cruciate ligament (ACL) reconstruction in animal models, highlighting its potential as a clinical strategy to promote healing at the tendon bone interface [130].

However, there is also conflicting evidence from studies. Tis et al. reported that while LIPUS increased the size of distracted callus and reduced fibrous tissue in a rabbit tibial osteotomy model, it had no positive effect on the mechanical properties or BMD of regenerated bone when applied during the consolidation phase—an outcome potentially attributable to suboptimal timing of application, late mechanical testing after initial callus differences had diminished, and a greater effect of LIPUS on callus quantity rather than quality [117]. Similarly, a canine study of 50 dogs with tibial plateau osteotomies found no significant differences in bone healing or limb use between the LIPUS and control groups, which may be attributable to insufficient sensitivity of radiographic assessment, limited sample size with low statistical power, and potential inhibition of LIPUS mechanisms by postoperative anti-inflammatory medication [119]. Furthermore, a randomized, blinded, controlled trial in a horse fourth metacarpal osteotomy gap model showed no effect of LIPUS on bone formation, potentially because the critical-sized gap exceeded the effective stimulation range of ultrasound [120].

#### 6.1.2. Osteoporosis Models

LIPUS also shows promise in osteoporotic animal models, which often use rats, mice, and rabbits. Studies report that it promotes bone formation in OVX rats, accelerates osteoblast differentiation, improves femoral defect healing, prevents estrogen-deficiency bone loss, and enhances bone volume/tissue volume (BV/TV), with significant increases in mineralized surface/bone surface ratios [18,123,124,125,126].

Sun et al. observed a strong correlation between LIPUS intensity (15–150 mW/cm^2^) and bone density, microstructure (R^2^ = 0.57 ± 0.83), and mechanical strength (R^2^ = 0.80 ± 0.97) in ovariectomized rats, with 150 mW/cm^2^ being most effective for bone mass maintenance—superior to the commonly used 30 mW/cm^2^ for fractures [56]. Wu et al. reported that LIPUS prevented bone loss in OVX rats, increased femoral wet weight, and improved the trabecular morphology [124]. Carvalho et al. observed more new bone formation and less microstructural deterioration in the proximal femoral cancellous bone of OVX rats who were treated with LIPUS [131]. A dose–response study demonstrated that 100 mW/cm^2^ LIPUS stimulation enhanced bone volume fraction, trabecular number, thickness, structural model index, mechanical strength, and apparent horizontal elastic modulus (a biomechanical property reflecting tissue stiffness in the horizontal plane) in osteoporotic rats, whereas 30 mW/cm^2^ only increased trabecular thickness [18]. Additional research showed that LIPUS reduces soft tissue pain and inflammation and promotes tissue repair [132]. Tang et al. found that LIPUS stimulation reduced myostatin (MSTN) levels in the quadriceps and serum of hindlimb unloading (HLU) rats, inhibiting its receptor and downstream signals while activating the Wingless/Integrated (Wnt) pathway in femurs, thereby preventing bone microstructure damage, maintaining mechanical properties, and promoting bone defect healing [133]. Yoshida et al. showed that LIPUS stimulation accelerated bone healing in low-turnover osteoporotic mice by promoting periosteal bone formation and compensating for reduced medullary bone formation [134], although the evidence in intact bones remains contradictory. Warden et al. found that ovariectomy-induced bone loss in rat hindlimbs was unaffected by LIPUS, which also had no effect on sham-operated rats, possibly due to cortical bone attenuation, insufficient ultrasonic energy, or a lack of signal entry points in intact bones [59,135]. Collectively, these studies demonstrate that LIPUS promotes bone formation and inhibits bone loss in osteoporotic models, although inconsistent results highlight the need for further research—especially on the effects of different intensities—across diverse osteoporotic animal models.

Despite its therapeutic potential, the application of LIPUS is constrained by inherent physical limitations, including significant signal attenuation due to reflection and scattering within bone—which may reduce its efficacy in stimulating deep tissues—as well as complex acoustic field distribution caused by skeletal heterogeneity and insufficient energy deposition resulting from the substantial acoustic impedance mismatch (approximately 2–3 fold) between trabecular and cortical bone [136,137]. To overcome these limitations, combining LIPUS with pharmaceutical agents or other physical modalities represents a promising strategy. For example, studies have shown that LIPUS synergizes with nanobubbles to enhance bone formation [138] and promotes dexamethasone- and/or transforming growth factor-beta1 (TGF-β1)-mediated chondrogenic differentiation of human mesenchymal stem cells [139]. Similarly, static magnetic fields (SMFs) have been demonstrated to inhibit bone loss, enhance osteogenesis, and improve bone microstructure and mechanical properties in various murine models of osteoporosis and fracture [140,141,142,143,144]. Our previous research further supports combinatory approaches, showing that SMF combined with Ferumoxytol mitigates unloading-induced osteoporosis in mice by reducing osteoclast activity and preserving bone mass [145]. Moreover, the concurrent application of SMFs and LIPUS generates the Lorentz force on charged particles (ions, cells, tissues), inducing rapid oscillations that produce localized currents and electric fields, while simultaneously optimizing ultrasound energy distribution through magnetic focusing to alleviate attenuation issues [146]. This combined approach provides both mechanical and electrical stimulation to bone tissue. Although existing studies indicate that magneto-acoustic coupling can enhance synaptic plasticity and inhibit cancer cell proliferation, its effects on the skeletal system remain unexplored [147,148]. Therefore, the combination of magnetic fields and ultrasound may represent a novel non-invasive therapeutic strategy for bone remodeling disorders such as osteoporosis.

### 6.2. Cellular Studies

Bone remodeling is regulated by osteoclast-mediated bone resorption and osteoblast-mediated bone formation, ensuring skeletal integrity and mineral homeostasis. As a dynamic tissue, bone undergoes continuous changes throughout life, governed by complex interactions among bone marrow mesenchymal stem cells, osteoblasts, osteoclasts, chondrocyte and osteocytes—each contributing uniquely to bone formation, resorption, and mechanotransduction [149,150]. Osteoblasts, derived from mesenchymal stem cells, are responsible for bone formation through extracellular matrix synthesis and mineralization, which are regulated by transcription factors like runt-related transcription factor 2 (RUNX2) and Osterix (Osx), which activate genes such as ALP and OCN [151,152]. Osteoclasts are multinucleated, hematopoietic stem cell-derived cells that mediate bone resorption by secreting acid and proteases. Their differentiation is regulated by M-CSF and RANKL and is additionally influenced by apoptosis and osteoblast signaling [153]. Embedded within the mineralized matrix, osteocytes act as mechanical sensors, coordinating adaptive responses to mechanical load through signaling pathways like TGF-β and Wnt/β-catenin [154]. Bone marrow mesenchymal stem cells (BMSCs) in the bone marrow niche serve as progenitors for osteoblasts and adipocytes, with differentiation being balanced by transcription factors such as peroxisome proliferator-activated receptor gamma (PPARγ) (adipogenesis) and bone morphogenetic protein (BMP)/sma and mad-related protein (Smad) (osteogenesis)—imbalances in which lead to pathologies like osteoporosis [155]. Endochondral ossification is critical for fracture healing, involving cartilaginous callus formation and its transition to bone. Failure in this transition leads to nonunion or delayed healing. Hypertrophic chondrocytes at the cartilaginous–bony callus interface regulate this process via two key mechanisms: (1) autocrine/paracrine signaling to coordinate matrix degradation, angiogenesis, osteoclast recruitment, and osteoblast differentiation; (2) direct transdifferentiation into osteoblasts to promote woven bone formation. Their role in bridging cartilaginous repair and bone remodeling highlights their indispensability in fracture healing [156].

To elucidate the biological mechanisms of LIPUS in bone remodeling diseases, researchers have focused on key cell types involved in bone remodeling: osteoblasts, osteoclasts, bone marrow mesenchymal stem cells, chondrocytes, and osteocytes.

#### 6.2.1. Osteoblasts

The current evidence indicated that LIPUS stimulation promoted osteoblast proliferation, differentiation, ALP activity, and mineralized nodule formation. For example, a 15 min LIPUS treatment enhanced cell viability and proliferation in mouse calvarial osteoblasts [19]. Similarly, LIPUS stimulation upregulated the expression of ALP and OCN during osteogenic differentiation in rat UMR-106 cells [157]. Gleizal et al. demonstrated that LIPUS stimulation significantly enhanced the expression of multiple osteogenic molecules in osteoblasts [158], and Hou et al. further elucidated that LIPUS treatment upregulated BMP-2 expression through specific signaling pathways [159]. Research also suggested that the effects of LIPUS on osteoblast function were both intensity-dependent and cell type-specific. Yang et al. found that LIPUS at intensities of 62.5, 125, and 250 mW/cm^2^ upregulated integrin α2, α5, and β1 expression in osteoblasts, with 125 mW/cm^2^ producing optimal effects in promoting mineralized nodule formation, increasing collagen content, and enhancing ALP activity [20]. Furthermore, LIPUS treatment accelerated mineralized nodule formation, enhanced ALP activity, and promoted early calcium deposition in human NHOst cells [124], while also increasing calcium accumulation in mouse MC3T3-E1 cells [160]. Mechanistically, LIPUS at 600 mW/cm^2^ stimulated proliferation by elevating intracellular Ca^2+^ levels and activating calmodulin [161]. However, in rat primary osteoblasts, although LIPUS stimulation did not induce extracellular Ca^2+^ influx, it still triggered transient upregulation of cellular friend leukemia virus one—specific oncogene (c-Fos), cyclooxygenase-2 (COX-2), IGF-Ⅰ, and OCN genes, suggesting cell type-specific responses [162]. A 10 min in vitro LIPUS treatment on osteoblast-like Saos-2 cells significantly increased osteoprotegerin (OPG) protein expression at 0 and 4 h post-treatment without affecting RANKL protein expression, indicating a rapid protein-level response [163].

As a key regulator of PGE2 synthesis essential for bone repair, COX-2 expression and PGE2 production were directly promoted by LIPUS exposure [164,165]. Tang et al. demonstrated that LIPUS exposure upregulated COX-2 and stimulated bone formation via activation of both the “integrin/FAK/PI3K/Akt” and “ERK” signaling pathways [166]. Co-culture experiments confirmed that LIPUS exposure enhanced osteoblast proliferation and ALP activity, supporting its pro-osteogenic effects [167]. Moreover, LIPUS treatment upregulated VEGF expression in osteoblasts, which promoted angiogenesis at fracture sites and provided vascular support for bone repair [168,169].

#### 6.2.2. Osteoclasts

Under specific parameter conditions, LIPUS stimulation was shown to inhibit osteoclast formation and differentiation. Studies demonstrated that daily 10 min LIPUS treatment at intensities of either 62.5 or 125 mW/cm^2^ applied between days 4 and 8 of osteoclast precursor differentiation suppressed osteoclastogenesis [20]. In a co-culture system of rat alveolar mononuclear cells and calvarial osteoblasts, LIPUS stimulation at 68 mW/cm^2^ over 7 days increased osteoblast numbers while decreasing osteoclast counts. It also elevated levels of TNF-α and PGE2—the latter being a potent inhibitor of multinucleated osteoclast formation—thereby achieving dual inhibition of osteoclast differentiation and bone resorption [167]. Subsequent investigation using FLG 29.1 osteoclast precursor cells revealed that LIPUS exposure inhibited the expression of cytoskeletal components and markers related to proliferation and differentiation, and also induced cellular damage [72]. It is well established that cytoskeletal depolymerization and reorganization represent typical cellular responses to gravitational and mechanical stimuli. Notable cytoskeletal alterations were found to cause redistribution and impairment of intracellular organelles, potentially leading to cell death [170]. These observations support the widely accepted hypothesis that the cytoskeleton mediates cellular gravity sensing and mechanical signal transduction [171,172]. Thus, the collective findings confirmed that prolonged mechanical stimulation via ultrasound could alter cytoskeleton-associated proteins in osteoclast precursors, ultimately inhibiting their differentiation.

#### 6.2.3. Bone Marrow Mesenchymal Stem Cells

BMSCs, the first isolated mesenchymal stem cells, are considered standard cells for clinical applications due to their proliferative and differentiation potential [173]. LIPUS exposure enhanced the viability and proliferation of BMSCs: Yang et al. reported a 19.57% increase in rat BMSC survival after LIPUS stimulation [174], while Xie et al. showed that LIPUS at 50 or 60 mW/cm^2^ promoted human BMSC proliferation through PI3K/Akt activation and cyclin D1 upregulation [175]. Additionally, Zhi and Aliabouzar observed increased proliferation as well as elevated collagen II and total collagen content in BMSCs following LIPUS treatment [176,177].

Numerous studies confirm that LIPUS influences BMSC differentiation. LIPUS treatment induced the transient upregulation of early response genes (including c-Jun, cellular myelocytomatosis oncogene (c-Myc), COX-2, early growth response–1 (Egr-1), and transforming growth factor-β-stimulated clone 22 (TSC-22)), as well as osteogenic markers such as ALP, osteonectin (ON), and osteopontin (OPN) in BMSCs within 3 h, suggesting a targeted effect on osteoblastic lineage cells [178]. Another study demonstrated that LIPUS enhanced intercellular communication in stromal cells via phosphorylation of ERK1/2 and p38, indicating a potential role in gap junction-mediated signaling [179]. Interestingly, lower LIPUS intensities (e.g., 2 mW/cm^2^) sometimes outperform clinical standards in boosting ALP activity and mineralization [180]. Multiple studies have indicated that LIPUS within a specific intensity range (20–50 mW/cm^2^) can significantly promote the chondrogenic differentiation of bone marrow mesenchymal stem cells by regulating the integrin/mammalian target of rapamycin (mTOR) signaling pathway and modulating autophagic activity [177,181,182]. In C2C12 cells, it upregulates osteogenic factors (RUNX2, p-ERK, p-p38) while suppressing adipogenic markers (CCAAT-enhancer-binding protein (C/EBP), PPARγ), favoring bone lineage commitment [183]. Kusuyama et al. showed that LIPUS exposure inhibits adipogenesis (reducing PPARγ2, fatty acid-binding protein 4 (FABP4)) and stimulates osteogenesis (increasing RUNX2, OCN) via ROCK-Cot-MEK-ERK signaling [184]. Li et al. found that high-intensity LIPUS (500–1500 mW/cm^2^) enhances HGF-induced hepatic differentiation in BMSCs via Wnt/β-catenin [185]. Another investigation demonstrated that LIPUS exposure exerts differential regulatory effects on the differentiation of human bone marrow mesenchymal stem cells (hBMSCs). The combination of LIPUS with dexamethasone/ TGF-β1 (TD) significantly potentiated chondrogenic differentiation, as evidenced by a transition to a rectangular/nodular morphology, substantial upregulation of chondrogenic markers (SRY-Box Transcription Factor 9 (Sox9), Aggrecan, and Collagen Type II (Col II), and increased Integrin β1 expression. Conversely, LIPUS monotherapy directly promoted osteogenic differentiation via upregulation of RUNX2 and Collagen Type I (Col I). When LIPUS is combined with BMP-2, cells exhibit a cuboidal osteogenic morphology, and the expression of osteogenic markers (RUNX2 and ALP) further increases, but the combined effect is not significantly additive. The study suggests that LIPUS selectively enhances TD-induced chondrogenic differentiation or BMP-2-driven osteogenic differentiation through the synergistic action of mechanical and biochemical signals. This implies that for clinical applications, LIPUS should be combined with specific inducers to optimize bone or cartilage repair, and different differentiation pathways may be related to integrin signaling and mechanotransduction mechanisms [139]. An et al. reported that 100 mW/cm^2^ LIPUS exposure upregulated osteogenic genes (Col I, ALP, OCN, BMP-2, OPN) and promoted osteoblastic differentiation in rat BMSCs [21]. These studies indicate that LIPUS exposure can regulate the differentiation of various stem cells into distinct lineages, offering adjuvant potential for subsequent stem cell therapies. Another investigation revealed that nanobubbles promote osteogenesis in BMSCs and LIPUS-induced bone formation by modulating transient receptor potential melastatin 7 (TRPM7), the actin cytoskeleton, and intracellular calcium oscillations. Additionally, reciprocal regulation between TRPM7 and actin filaments enhances the synergistic effects of LIPUS/nanobubbles [138]. Such findings provide new directions for optimizing the efficacy of LIPUS in fracture healing, suggesting promising combinations with other pharmacological or physical therapies to establish a theoretical foundation for its clinical translation and advancement. Conversely, Naruse et al. exposed bone marrow-derived stromal cells to LIPUS and found them to be largely insensitive, attributing observed data discrepancies to variations in LIPUS exposure timing [186].

While effects of LIPUS on BMSC viability, proliferation, and differentiation are well-documented, mechanistic studies remain in their infancy, with most research being limited to in vitro models. Future efforts should prioritize in vivo validation, clinical trials for LIPUS-BMSC combination therapy, and standardizing parameters (frequency, intensity, duration) to optimize therapeutic outcomes.

#### 6.2.4. Chondrocytes

A study has shown that LIPUS at intensities ranging from 0.1 to 0.77 W/cm^2^ promotes chondrocyte proliferation [187]. In a rabbit patellar tendon reconstruction model, LIPUS stimulation increased VEGF expression in the chondrocytes of woven bone, enhancing tissue repair [168]. Furthermore, LIPUS facilitates chondrocyte differentiation: experiments using C2C12 cells showed that it upregulates Sox9 mRNA expression and activates the ERK1/2/p38 MAPK signaling pathway during chondrogenesis, thereby promoting differentiation toward a chondrogenic phenotype [183]. A pellet culture study further revealed that LIPUS stimulation promoted chondrocyte proliferation, maintained differentiation, and required TGF-β1 for its effects [188]. Additionally, low-intensity ultrasound exposure also upregulated aggrecan gene expression in rat femoral fracture models, supporting cartilage matrix maintenance [189].

#### 6.2.5. Osteocytes

LIPUS stimulation of osteocytes derived from neonatal rat tibiae and calvariae upregulated the expression of c-Fos and COX-2 but did not affect IGF-I or osteocalcin, and no extracellular Ca^2+^ influx was detected [162]. Exposing osteocytes to LIPUS at three axial distances (0 mm, 60 mm, 130 mm) and then using their conditioned medium to culture pre-osteoblasts revealed widespread nuclear and perinuclear β-catenin signals in the 60 mm and 130 mm groups, indicating that osteocytes sense acoustic differences at varying distances. Far-field (130 mm) LIPUS-exposed osteocytes promoted pre-osteoblast migration, maturation (proliferation to differentiation), and matrix calcification via paracrine factors, highlighting the role of osteocytes in transmitting mechanical signals to osteoblasts [190]. This finding demonstrates the positive effect of far-field LIPUS in stimulating bone cells and facilitating the mechanical transduction between bone cells and osteoblasts. Shimizu et al. demonstrated in vivo that osteocytes enhance fracture repair mediated by LIPUS through the transcriptional upregulation of Egr1, Egr2, FoxQ1, Helz, JunB, and NFATc1—genes that were identified in this study as osteocyte-specific mechanotransduction targets that are responsive to LIPUS. This transcriptional activation induces target gene-mediated cellular metabolism and survival while activating key signaling pathways that are essential for fracture healing, including TGF-β and Wnt-dependent cascades [191]. These findings demonstrate that LIPUS primarily modulates bone cells through the mechanosensory function of osteocytes, which indirectly influences osteoblastic and osteoclastic activities via mechanotransduction pathways.

**Figure 4:**
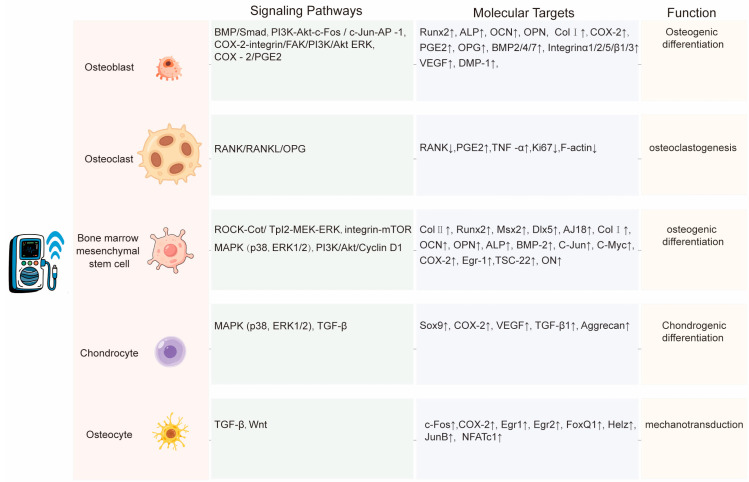
Signaling pathways and molecular targets of LIPUS for influencing osteogenic differentiation of BMSCs, osteogenic differentiation of osteoblasts, chondrogenic differentiation, osteoclastogenesis, and mechanotransduction of osteocytes. This schematic diagram summarizes the main signaling pathways and molecular targets involved in the regulation of key biological processes of bone-related cells by LIPUS, as well as their expression trends. ↑ denotes upregulation, and ↓ denotes downregulation.

In conclusion, ultrasound acts on bone cells through various molecular and signaling pathway routes (Figure 4). These molecules and pathways also influence each other and regulate bone remodeling. Additionally, at the cellular and animal levels, an increasing number of studies have identified other therapeutic approaches for regulating bone remodeling, such as electromagnetic field therapy, gene therapy, and drug therapy, apart from the molecular signaling pathways mentioned earlier. These findings provide new insights into the clinical application of LIPUS. For example, combining LIPUS with other multi-physical fields or using it in combination with inhibitors, drugs, etc., can offer a new perspective for studying the biological mechanisms of the effects of LIPUS on bone remodeling.

## 7. Physical Mechanisms of LIPUS on Bone

Since the 1990s, when LIPUS was approved by the U.S. FDA for clinical fracture treatment, research on ultrasound-mediated bone remodeling has evolved into a dual paradigm encompassing biochemical and biophysical investigations. Recent advancements in molecular and cellular biology have deepened our understanding of LIPUS’s biochemical mechanisms in bone tissue. However, as a unique form of mechanical stimulation, the energy transfer and conversion mechanisms of ultrasonic mechanical vibrations within biological tissues remain incompletely resolved, leading to gaps in the scientific explanation of LIPUS’s therapeutic effects. Current research urgently requires breakthroughs in deciphering the multi-scale coupling of “acoustic wave–mechanical/electrical/thermal signals–tissue/cell” interactions.

By treating biological systems as specialized materials and leveraging the inherent acoustic properties of tissues, this section analyzes the secondary physical quantities that are generated by LIPUS in bone tissue, including mechanical, piezoelectric, and thermal signals. These multi-physical interactions directly drive molecular and cellular responses, forming a complete chain of “acoustic stimulation–mechanical/electrical/thermal signaling–biological response”. Critically, ultrasound–tissue interactions produce three primary biological effects: mechanical effects at bone–soft tissue interfaces, piezoelectric effects in bone, and thermal effects. Establishing multi-physics coupling models to systematically investigate the “acoustic field–electro-thermo-mechanical microenvironment–cellular response” biophysical mechanisms will define a new paradigm for therapeutic LIPUS research.

### 7.1. Mechanical Effects

Ultrasound refers to mechanical vibration waves with frequencies >20 kHz, which are inaudible to humans. As these waves propagate through a medium, they induce periodic oscillations of particles, generating dynamic pressure changes that produce significant mechanical effects. These effects include increased cell volume, altered membrane permeability, enhanced metabolite exchange, and regulation of cellular functions, with applications in treating neural, muscular, and skeletal injuries [45,50]. The mechanical effects of LIPUS primarily manifest as acoustic radiation force, acoustic streaming, and acoustic cavitation [192]. For example, ultrasound at 0.8 MHz and 2 W/cm^2^ can generate pressure waves equivalent to 2.6 atmospheres [64]. LIPUS delivers low-intensity mechanical stimuli to cells, inducing biochemical responses that promote tissue repair and regeneration [86]. Such stimuli transiently modulate cell/tissue permeability, trigger pro-inflammatory/proliferative reactions, alter genetic activity, and influence neuronal signaling [193].

#### 7.1.1. Acoustic Radiation Force

The acoustic radiation force (ARF) arises from pressure gradients in an ultrasonic field [194]. In LIPUS studies, the ARF (*F_rad_*) is calculated as follows:

(1) $$F_{rad}=\frac{W}{c_{0}}$$

where *W* is the total acoustic output power (W) and *c*_0_ is the speed of sound (m/s). The ARF typically ranges from micronewtons (μN) to millinewtons (mN) and can be measured via ultrasonic power balances [45]. For instance, 1 mW of acoustic power in water generates 0.69 μN of force [195]. In bone tissue, ARF-induced periodic pressure changes convert mechanical energy into cellular displacements and vibrations [196,197], activating mechanosensitive receptors and signaling pathways (e.g., cell proliferation, differentiation, and osteogenesis).

#### 7.1.2. Acoustic Streaming

Acoustic streaming refers to fluid flow or tissue motion caused by momentum transfer in liquid or soft tissue media [198]. It enhances circulation, nutrient diffusion, and molecular redistribution [45]. Pressure gradients from acoustic attenuation generate time-averaged forces, creating micro-vortices [199]. The force generated by the conversion of acoustic energy attenuation into fluid momentum is the driving force of acoustic streaming, and its calculation formula can be simplified as follows:

(2) $$F\approx \frac{2\alpha }{c}I$$

where *F* is the driving force (N), *I* is the acoustic intensity (W/m^2^), *c* is the speed of sound (m/s), and *α* is the sound attenuation coefficient (Np/m). Flow patterns depend on boundary conditions (e.g., bone surfaces reflecting waves to form standing waves or vortices) [200]. Laser Doppler anemometry (LDA) or particle image velocimetry (PIV) can be used to quantify streaming [201,202]. Shear stress from streaming activates mechanotransduction pathways, influencing osteoblast/osteoclast differentiation and bone remodeling [42,67].

#### 7.1.3. Acoustic Cavitation

Cavitation refers to the oscillatory growth, shrinkage, and collapse of microbubbles in liquid media due to periodic pressure changes during ultrasound propagation [203] and exists in two forms: stable cavitation and inertial cavitation [42]. Ultrasonic intensities < 100 mW/cm^2^ rarely induce inertial cavitation but may generate stable cavitation microflows [204,205]. LIPUS primarily exhibits stable cavitation in experimental studies, where bubbles oscillate periodically under sound pressure (without violent collapse), producing local microflows, shear forces, and mechanical stress [206]. The cavitation intensity depends on parameters like the duty cycle, frequency, sound pressure, pulse length, and PRF [207]. Low-frequency ultrasound (e.g., 25 kHz) primarily induces cavitation, while high-frequency ultrasound (e.g., 2 MHz) generates stable Eckart streaming—a steady circulatory pattern in fluids [208]. Cavitation affects physiological responses via liquid oscillation or direct tissue interaction, which can be observed using high-speed photography or acoustic emission sensors [209,210]. Theoretical models (e.g., the Rayleigh–Plesset equation) combined with sound propagation equations simulate cavitation effects [210]. In protein-containing media, ultrasound–protein interactions via mechanical and cavitation effects alter protein properties (foamability, solubility, gelation), although excessive cavitation may cause tissue damage, hemorrhage, intravascular hemolysis, or cell death, depending on the intensity [211,212].

### 7.2. Piezoelectric Effect

While most studies attribute the effects of LIPUS to mechanical actions, we emphasize the critical role of the piezoelectric effect in bone remodeling. The piezoelectric effect of LIPUS occurs when ultrasonic mechanical forces act on bone tissue, inducing polarized charges due to the non-centrosymmetric structure of collagen fibers and hydroxyapatite crystals [213]. In 1962, Bassett CA observed that mechanical stress on bone generated negative potentials in compressed regions, a phenomenon that was later termed the bone piezoelectric effect, consistent with findings by Fukada E and Yasuda I [213,214]. Then, in 1964, Bassett CA demonstrated that implanted electrodes promote new bone growth in negative potential regions, first proposing electrical stimulation for osteogenesis [215]. External electrical stimulation is invasive and carries risks of electrolytic effects and infection, limiting its use for most skeletal diseases [216]. In contrast, LIPUS is non-invasive and generates piezoelectric potentials to mimic electrical stimulation for bone formation. Suzuyama et al. simulated ultrasonic wave propagation and induced potentials in bone using piezoelectric finite-difference time-domain methods, confirming ultrasonically induced potentials in heterogeneous bone structures that were comparable to those from electrical stimulation [217]. Hosokawa numerically modeled the piezoelectric effect in human femurs under ultrasound, showing electric field variations with bone structure, and detected piezoelectric signals in trabecular bone specimens that were exposed to 1 MHz ultrasound [136,218], confirming that ultrasonic mechanical forces generate piezoelectric electromotive forces in bone. Piezoelectric potentials in bone can be detected using microelectrode arrays or scanning Kelvin probe microscopy for surface potential mapping, but the internal potential distribution remains challenging to measure, requiring new simulation methods [219]. Bone is a natural piezoelectric material with a longitudinal piezoelectric coefficient d_33_ of 4–11 pC/N [220]. For the propagation of lossless linear plane waves in ultrasound, the calculation formula for acoustic pressure is as follows:

(3) P=ρcv

where *P* is the acoustic pressure (Pa), *ρ* is the medium density (kg/m^3^), *c* is the sound speed (m/s), and *v* is the particle vibration velocity (m/s). The constitutive equation of the piezoelectric effect is expressed as follows:

(4) $$D_{j}=d_{jkl}^{d}\sigma_{kl}+\varepsilon_{jk}^{\sigma }E_{k}$$

where *D*_*j*_ is the electric displacement (C/m^2^), *E*_*k*_ is the electric field (V/m), *σ*_*kl*_ is the mechanical stress (Pa), *ε*^*σ*^_*jk*_ represents the dielectric constant (F/m) under constant stress, and *d*^*d*^_*jkl*_ is the piezoelectric voltage constant (C/N). When the electric field effect is ignored (*E* = 0), the piezoelectric effect of ultrasound on bone tissue can be simplified as follows:

(5) D=d⋅σ

where *σ* is the acoustic pressure generated by ultrasound (Pa), *d* is the piezoelectric constant of bone tissue (pC/N), and *D* is the electric displacement (C/m^2^). In a non-uniform medium, it also depends on the position in the medium [221]. The above calculation is the theoretical value in a static field. In practice, dynamic ultrasound may enhance the local electric field through charge accumulation or the capacitance effect of the cell membrane. A study showed that the maximum electric field strengths that can be obtained by simulating the piezoelectricity generated by ultrasonic exposure on the female radius are 4.3 mV/cm and 5.6 mV/cm [217].

### 7.3. Thermal Effects

While mechanical effects are widely recognized as the primary biological mediators of LIPUS, thermal effects—although negligible at low intensities—become significant at higher levels. The thermal effect of ultrasound arises from energy absorption (viscous and relaxational losses) in biological tissues, converting acoustic energy to heat via molecular friction, which elevates the local temperature and affects cell excitability [222]. An excessive temperature denatures enzymes and proteins, reducing biological activity [223]. Thermal changes depend on intensity, exposure time, and tissue properties (absorption coefficient, thermal conductivity). Higher absorption coefficients, intensities, or durations enhance thermal effects. Infrared thermography or thermocouples measure temperature changes in experiments [224]. Clinical LIPUS (intensities < 500 mW/cm^2^) rarely induces biologically significant heating, but high-intensity ultrasound (5000–25,000 mW/cm^2^) causes adverse effects like healing arrest, fibrosis, and necrosis in dog femurs [225]. A 2400 mW/cm^2^ pulsed ultrasound exposure for 15 min only induced a 0.2 °C medium temperature rise, measured via fine-wire thermocouples [161]. High-intensity focused ultrasound (39–65 W power) causes soft tissue damage and osteocyte necrosis in rabbit femurs, disrupting bone and cell metabolism [226]. Notably, a single study reported that 30 mW/cm^2^ LIPUS application for 30 min promoted osteoblast proliferation/differentiation via heat-sensitive protein (HSP70) regulation, despite minimal temperature elevation (37 °C to 40 °C), suggesting that cells detect minor thermal changes and mount subcellular responses [70].

The physical mechanisms of LIPUS involve complex interactions between acoustic energy and biological tissues, with mechanical, piezoelectric, and thermal effects acting in concert to regulate influence bone remodeling. While mechanical effects dominate at clinical intensities, piezoelectric signaling and subtle thermal cues may modulate cellular responses. Decoding the synergistic contributions of these physical factors—especially through multi-physical modeling and in vivo validation—represents a pivotal frontier for optimizing LIPUS therapy and advancing its clinical translation.

### 7.4. Interactions Between LIPUS-Generated Secondary Physical Quantities and Biological Tissues

The ultrasonic biological effect generally refers to the physiological and biochemical changes that occur at multiple levels, such as the whole organism, cells, and molecules, when ultrasound acts on a living organism. Usually, the general parameters of ultrasound (intensity, frequency, power, time, etc.) are regarded as the “cause”, which is associated with the indicators of biological changes as the “result”. Taking bone tissue as an example, which physical factors act on it during the process of ultrasound exposure? Through which “physical factor–biochemical coupling” processes do these physical factors produce biological effects? To answer these questions, we need to consider that the externally applied ultrasound generates various “secondary physical quantities” through the force-electrical properties of bone tissue itself, and these quantities then act on the bone tissue, causing relevant biological, biochemical, and physiological effects [221].

Bone tissue is composed of an extracellular matrix that is rich in organic collagen fibers, various proteins such as osteocalcin, and a small amount of potassium, magnesium, calcium salts, and inorganic hydroxyapatite, as well as cellular components, including bone marrow mesenchymal stem cells, osteoblasts, osteocytes, and osteoclasts [227,228,229]. The minerals, collagen, and protein ions that are adsorbed on the surface of bone and cells form the growth environment of bone. The composition of bone tissue and its growth environment determine its electrophysiological properties: its piezoelectricity, streaming potential, and bioelectric potential [230,231]. Piezoelectricity means that when bone tissue is under pressure, the sliding between collagen fibers generates a piezoelectric voltage [230]. The streaming potential refers to the fact that the potential in bone is caused by the separation of surface charges due to the liquid flow that is induced by stress, forming an electric double layer. When the fluid flow disrupts the double layer, an electric current is generated, thus forming a streaming potential, also known as the zeta potential, between the two electrodes [231]. Bioelectric potential means that osteogenically active regions, such as bone marrow, are electronegative (relative cathode) compared to inactive regions, which is jointly generated by metabolic and biochemical actions [231]. The piezoelectric and streaming potentials of bone tissue are called stress-generated potentials (SGPs) [232]. Normal physiological activities, such as jumping and running, apply mechanical loads to bone tissue, stimulate bone formation, and promote structural changes in bone tissue [233]. Even under normal static conditions, bone tissue in the body is constantly subjected to mechanical stimuli from various sources, including gravity (macro-level) and blood circulation (micro-level) [234]. Mechanical stimuli are part of the bone tissue microenvironment and are crucial for maintaining bone health and dynamic balance [235]. Bones can adjust their structure and morphology according to the mechanical loads they bear, which is considered the main mechanism behind Wolff’s law. Most of the electrical properties of bone tissue are related to external loads and strains, especially the piezoelectric effect and the streaming potential effect, which are collectively referred to as the stress-generated potentials.

As mentioned before, ultrasound can generate acoustic radiation force, acoustic streaming, cavitation, thermal effects, and piezoelectricity in bone tissue. The force and electrical signals among them will interact with the SGP of the bone tissue itself. We believe that the multiple secondary physical quantities of “acoustic/mechanical/thermal/electrical” effects that are generated by ultrasound in bone tissue greatly influence the process of bone remodeling and metabolic balance by affecting the molecular motion of bone tissue and cells and the transduction of signal pathways. Mechanosensitive (MS) ion channels refer to a group of transmembrane channel proteins that can convert mechanical stimulus signals into electrical or chemical signals [223]. In osteoblasts, LIPUS-mediated mechanotransduction primarily occurs through the mechanosensitive channels Piezo1 and Trpv4. Activation of Piezo1 triggers calcium influx and ERK phosphorylation, promoting cellular proliferation. Trpv4 synergistically enhances calcium-dependent MAPK signaling with the big-conductance calcium-activated potassium channel (BKca), although its specific role in osteoblast mechanotransduction requires further investigation [236]. The current evidence for these pathways is primarily derived from in vitro models, necessitating future studies to validate their functions within physiological contexts. As a mechanical wave, ultrasound exerts a radiation force on biological tissues. The acoustic radiation force can cause mechanical vibrations and cell membrane deformations, activate mechanosensitive ion channels in neurons, and make cells discharge [237]. Potassium channels are a diverse group of proteins that play a crucial role in regulating cell activity and maintaining cell homeostasis. Two-pore-domain potassium channels with the ability to sense mechanical stimuli on the cell membrane are classified as mechanosensitive channels, namely, TWIK-related K^+^ channel-1 (TREK-1), TREK-2, and TWIK-related arachidonic acid-activated K^+^ channel (TRAAK) [223]. A study found that the application of short-pulse, low-intensity ultrasound (5 MHz, 1.2 W/cm^2^,10 ms) led to the rapid and strong activation of the TRAAK in the patches of Xenopus oocytes expressing TRAAK and in the cortical neurons of mice also expressing TRAAK, while the non-mechanosensitive two-pore-domain potassium (K2P) channels were not activated by ultrasound [238]. Another study showed that ultrasound can indeed generate measurable mechanical forces. When exposed to a hydrogel encapsulating MC3T3 mouse osteoblasts, it can cause the hydrogel to deform and promote an increase in the gene expression of alkaline phosphatase and osteocalcin in osteoblasts, indicating that the mechanical force provided by ultrasound may, in part, induce bone formation by physically stimulating cells [239]. Most studies on the biophysical mechanisms of LIPUS treatment mainly focus on the cell membrane, where acoustic stimuli are converted into biological signals [240]. LIPUS can deform the lipid membrane tension through the acoustic radiation force or the direct mechanical interaction between the membrane and sound waves [228,241], stimulating mechanosensitive receptors and channels to trigger a signal cascade reaction. A study showed that the process of insulin secretion in pancreatic β-cells stimulated by 800 kHz, 0.5 W/cm^2^ LIPUS was proven to be a Ca^2+^-dependent mechanism. This mainly occurred through transient membrane permeabilization (a process that is considered to be mostly mediated by acoustic cavitation) to allow Ca^2+^ entry, thus triggering the release of the contents of secretory vesicles through exocytosis [242]. In contrast to the cell membrane, the interaction between LIPUS and the intracellular domain is hardly considered as a potential mechanism. However, another study attempted, for the first time, to reveal the physical connection between LIPUS and the dynamics of intracellular macromolecules: LIPUS may increase the diffusivity of intracellular macromolecules through sound pressure and mechanical stimulation, resulting in changes in basic cell processes, namely, an increase in the diffusion rate and phosphorylation of passive nucleocytoplasmic transport and extracellular signal-regulated kinase ERK. Our research results can provide a new perspective that, in addition to the cell membrane, the intracellular region can also convert the acoustic stimuli of LIPUS into a mechanical transduction process of biochemical signals [192]. Numerous studies have confirmed the hypothesis that osteocytes are mechanical sensors. They can sense the strain on the bone surface through stretch—activated ion channels, interstitial fluid flow, and electrical potential, and thus generate signals in response to mechanical loads [243]. The continuous remodeling of normal bone tissue, the development of new bone, and the repair of traumatic bone defects are strongly regulated by physiological stimuli and external mechanical forces [244].

Existing studies have shown that 1 MHz ultrasonic exposure can generate piezoelectric signals in cancellous bone specimens, indicating that ultrasound generates piezoelectric potential in bone. Piezo1 is a mechanosensitive piezoelectric ion channel that can respond rapidly to various forms of mechanical stimuli and convert mechanical signals into biochemical signals to regulate various physiological processes. Some studies have shown that the activation of Piezo1 plays a crucial role in the mechanical transduction of ultrasonic stimulation. Qiu et al.’s research showed that low-intensity and low-frequency ultrasonic stimulation can activate the endogenous Piezo1 channels in HEK293T cells, trigger Ca^2+^ influx, and increase the expression level of nuclear c-Fos in primary neurons. This effect can be inhibited by pretreatment with a Piezo1 inhibitor. In addition, ultrasonic stimulation significantly affects the levels of downstream Ca^2+^ signaling proteins, inducing the expression of important proteins such as phospho-Calcium protein kinase II (CaMKII), phospho-Cyclic adenosine monophosphate response element-binding protein (CREB), and c-Fos in neuronal cell lines. These proteins play important roles in complex neuronal functions such as learning, memory, and neuronal synaptic plasticity [245]. Another study showed that compared with the control group, the knockout of Piezo1 in the right motor cortex of mice significantly reduced the ultrasonically induced neuronal calcium response, limb movement, muscle electromyogram signaling, and c-Fos expression [241]. Piezo1 is expressed in both neurons and astrocytes. Research has proven that Piezo1 is expressed in different brain regions, and Piezo1 in neurons plays an important role in directly mediating the effects of ultrasound; the expression level of Piezo1 in neurons of the central amygdala (CEA) of the brain is more sensitive to ultrasound than cortical neurons [246]. A study showed that Piezo1 can sense the mechanical signals induced by LIPUS, allowing extracellular calcium ions to enter the cell. The influx of Ca^2+^, as a second messenger, activates the phosphorylation of ERK1/2 and the polymerization of perinuclear F-actin filaments, regulating the proliferation of MC3T3-E1 cells [247]. At the same time, some studies have also preliminarily demonstrated the mechanism of electro-stimulation-induced osteogenesis: electrical signal transduction can increase bone-forming proteins, β-growth factors, and insulin-like growth factor II [248]. Electrical stimulation can change the membrane potential and other factors, thus trigger physiological reactions in cells. It can also improve the local blood environment to create an osteogenic micro-environment and promote bone formation [249]. Some studies have also found that coupled electrical stimulation can effectively increase morphogenetic proteins and promote bone marrow fusion [240]. Based on the summary and analysis of a large number of clinical reports, Aaron et al. also concluded that electrical stimulation can promote bone healing, and that its effect on bone healing and repair in osteotomy, delayed bone union, and nonunion is more obvious [250].

Regarding the thermal effect of ultrasound, only an excessively high intensity is harmful to cells. A study showed that when the average threshold level of ultrasonic intensity is greater than 5.7 W/cm^2^ and the exposure time exceeds 3 min, it can lead to the death of glioma cells [251]. A study showed that 43 MHz, 50 W/cm^2^ ultrasound can activate thermosensitive and mechanosensitive K2P channels through the heating or mechanical effect induced by the acoustic radiation force. The finite element model of the effect of ultrasound on brain tissue shows that ultrasound can affect the discharge frequency of brain tissue by slightly increasing the temperature (<2 °C) and possibly through mechanical effects [252]. In contrast to high-intensity continuous ultrasound, LIPUS is a pulsed emission with low intensity and low thermal effect, causing little or no adverse effects on cells [253]. Bader et al. believed that LIPUS is not sufficient for producing a significant thermal effect, but it may cause tissue displacement or fluid flow due to the radiation force [254]. Another study showed that an intensity of 50 mW/cm^2^ will not cause a temperature change greater than 1 °C [255,256]. Ferreri et al. used an ultrasonic signal of 100 mW/cm^2^, which is lower than the intensity known to induce thermal heating and considered safe for the treatment of humans and animals [18]. Currently, most of the known experimental and clinical applications use an ultrasonic intensity of less than 100 mW/cm^2^, which has no significant risk of thermal damage to bone tissue. Its therapeutic effect mainly depends on the mechanical and piezoelectric effects. Heat shock protein (HSP) is a protein that is ubiquitous and highly conserved in all organisms under various stress conditions. Its synthesis can be triggered not only by heat shock but also by several physical stimuli. Currently, some studies have also shown that exposing cells to 30 mW/cm^2^ LIPUS for 30 min may promote the osteogenic differentiation of human adipose-derived stem cells in vitro by regulating the expression of the intracellular thermosensitive proteins HSP70 and HSP90. This temperature increase was able to induce HSP expression, indicating that the thermal effects of LIPUS can be detected by cells and lead to a sub-cellular-level response [70]. Further research could be carried out to explore whether a micro-thermal effect (such as 0.5–1 °C) can be generated by appropriately increasing the ultrasonic intensity to synergistically enhance the healing-promoting effect of mechanical signals. An international unified safety guideline for ultrasonic treatment needs to be established for clinical transformation standards. The influence of the ultrasonic thermal effect is highly complex and related to many factors, which indicates that in clinical applications or related research, the intensity, action time, and other parameters of ultrasound must be precisely controlled. For example, in bone-related treatments, choosing appropriate ultrasonic parameters can not only play a positive role of ultrasound, such as using LIPUS to promote certain physiological processes in bone tissue, but also avoid potential hazards caused by thermal effect. At the same time, in the current research, significant room remains for research on the differences in the responses of different types of cells (for example, only glioma cells are mentioned) and different tissues (although bone tissue is considered safe, more in-depth and detailed research is lacking) to the ultrasonic thermal effect. In the future, it may be possible to further explore the specific molecular mechanism of the ultrasonic thermal effect on bone tissue cells, as well as explore ways to more optimally adjust the ultrasonic parameters according to individual differences to achieve a safer and more effective treatment effect. In addition, the long-term impact of potential tissue displacement or fluid flow caused by acoustic radiation force on the normal physiological functions of bone tissue needs to be further evaluated.

**Figure 5:**
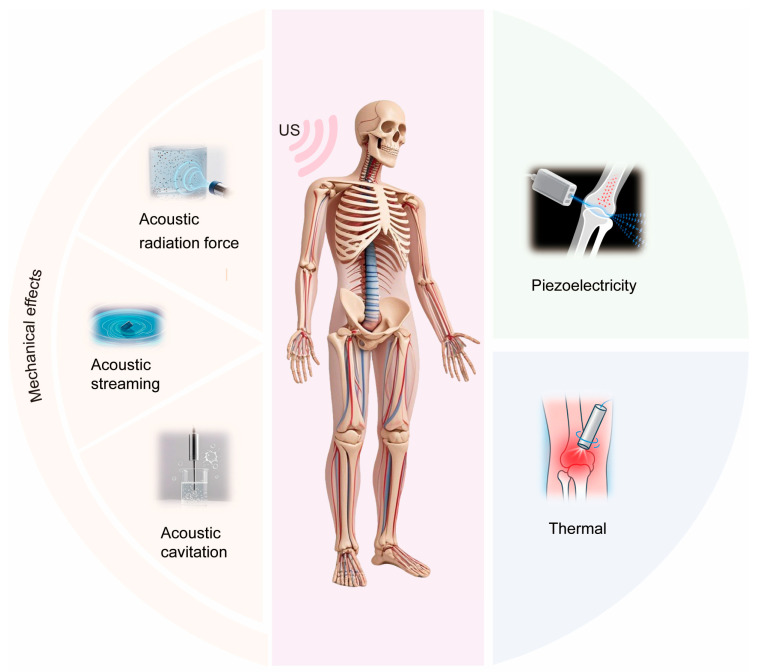
Physical mechanism of LIPUS in bone remodeling. Upon interacting with bone tissue, LIPUS elicits three primary physical effects: mechanical effects (acoustic radiation force, acoustic streaming, acoustic cavitation), piezoelectric effects, and thermal effects.

In conclusion, when tissues and cells are in an ultrasonic field with specific parameters, the ultrasound generates multiple secondary physical effects such as “acoustic/mechanical/electrical/thermal” processes inside the cells, which then trigger biological effects from the molecular and cellular levels to the whole organism. This is the physical mechanism of impact of ultrasound on living organisms (Figure 5).

## 8. Conclusions and Future Perspectives

LIPUS, as a non-invasive therapeutic modality, has demonstrated efficacy in promoting fracture healing since the 1990s, and has since been extended to the treatment of bone remodeling-related disorders, including nonunion and osteoporosis. By converting acoustic energy into biomechanical, electrical, and thermal signals through mechanical, thermal, and piezoelectric effects, LIPUS influences bone cell metabolism and microenvironmental remodeling. However, critical gaps persist in understanding its underlying biophysical mechanisms, particularly the integration of “acoustic/mechanical/electrical/thermal” multi-physical field coupling effects and their synergistic regulatory dynamics. The current research predominantly focuses on superficial correlations between ultrasound parameters (e.g., frequency, intensity, exposure time) and biological outcomes, while the understanding of how mechanically induced piezoelectric effects activate bone cell functions through signal transduction remains highly limited. Disease-specific mechanisms, such as differential responses in osteoporotic fractures versus nonunion, also remain poorly defined. Furthermore, the absence of a well-established dose–effect relationship necessitates empirical parameter selection in clinical practice, hindering personalized therapeutic strategies. Systematic exploration of critical parameters—including the duty cycle, pulse duration, and working distance—is urgently required to optimize treatment precision.

Technologically, conventional LIPUS devices rely on legacy parameters (e.g., 1.5 MHz, 30 mW/cm^2^), which, while safe, exhibit variable efficacies due to interpatient heterogeneity. Emerging approaches, such as high-frequency ultrasound (>3 MHz) and composite pulse modulation, hold promise for targeted bone regulation but remain in exploratory phases. Current systems are further limited by bulky designs, prolonged treatment protocols (20–40 min daily for 3–6 months), and insufficient intelligence integration, resulting in poor patient compliance and clinical outcomes. Although innovations like wearable ultrasound devices demonstrate preclinical potential, challenges persist in terms of energy stability, long-term safety, and clinical translation. The lack of real-time biofeedback and adaptive parameter adjustment capabilities further underscores the need for advancements in intelligence and portability to adapt interventions to dynamic bone healing processes.

To address these challenges, a multidisciplinary roadmap is proposed:

- Mechanistic Elucidation: Multi-omics platforms (transcriptomics, proteomics) should be integrated with multi-physical field modeling (e.g., finite element simulations) to establish multi-scale systems biology frameworks. These models should quantify the synergistic interplay of acoustic pressure, mechanical stress, piezoelectric potentials, and localized thermal changes induced by LIPUS. For example, 3D bone organoid systems replicating in vivo mechanical microenvironments, coupled with single-cell sequencing and validated through biomolecular experiments, could delineate dose–effect relationships between LIPUS parameters and repair outcomes.
- Technological Innovation: Artificial intelligence (AI) and advanced ultrasound engineering should be leveraged to develop precision therapeutic strategies. Machine learning-driven analysis of clinical datasets could optimize high-frequency, narrow-pulse protocols (e.g., 3 MHz, 50 μs) to mitigate thermal risks. Hybrid therapies combining LIPUS with pharmacological agents or electromagnetic fields may overcome the inherent limitations of single-modality interventions.
- Clinical Translation: The development of miniaturized transducers and energy-efficient systems should be accelerated to enable intelligent wearable devices. Integration of biosensors (e.g., impedance monitoring, optical coherence tomography) will facilitate real-time biofeedback and adaptive parameter modulation. Concurrently, telemedicine-compatible home-use devices could revolutionize chronic bone disease management, bridging the gap between laboratory innovation and community healthcare delivery.

In conclusion, while LIPUS represents a transformative approach to bone remodeling, its full potential hinges on resolving fundamental biophysical questions, refining parameter optimization, and developing intelligent device architectures. Future endeavors must foster cross-disciplinary collaboration to dissect the individual and collective contributions of multi-physical stimuli, establish evidence-based parameter standards, and expedite the translation of intelligent therapeutic platforms. These advancements promise to deliver precise, equitable, and minimally invasive solutions for bone repair, ultimately enhancing patient outcomes worldwide.

## Figures and Tables

**Table 1:** Details on the parameters of LIPUS used in bone remodeling disorders.

Parameters	Intensity	Frequency	Pulse Repetition Frequency	Pulse Width	Pulse Duty Cycle	Exposure Time
Value	30 mW/cm^2^	1.5 MHz	1 kHz	200 μs	20%	20 min

**Table 2:** Three types of ultrasound intensity.

Acronym	Definition	Physical Meaning	Calculation Formula (Example)	Symbol and Description
I_sppa_	The average intensity at the strongest point in the beam within a single pulse.	Represents the instantaneous peak intensity of a pulse, directly related to the peak pressure amplitude.	$I_{sppa}=\frac{{p_{\mathrm{sp}}}^{2}}{2Z}$	p_sp_: Spatial-peak pressure amplitude (Pa) Z: Characteristic acoustic impedance (Rayl)
I_spta_	The average intensity at the strongest point in the beam over the total exposure time (including pulse off periods).	Reflects the average energy level at the beam’s hotspot over prolonged exposure; the primary determinant for thermal effects.	$I_{spta}=I_{\mathrm{sppa}}DC$	DC: Duty cycle
I_sata_	The average intensity over the entire beam cross-sectional area and the total exposure time.	Represents the average power density across the entire transducer output face.	$I_{sata}=\frac{W}{a}$	W: Acoustic output power (W) a: Transducer surface area (m^2^)

**Table 3:** Details of clinical studies on the effect of LIPUS on bone remodeling disorders.

Ref	Number of Cases	Target	Parameters (F, I_SATA_, PD, PRF, PDC, T) *	Treatment Period (days)	Positive Results	Negative Results
[87]	51	Rib fractures	1.0 MHz, 500 mW/cm^2^, N/A, N/A, 10%, N/A	180	This study presents the first evidence that LIPUS treatment is capable of improving rib fracture outcome, significantly accelerating bone callus healing and decreasing pain.	N/A
[88]	30	Fresh scaphoid fractures	1.5 MHz, 30 mW/cm^2^, 1 kHz, 200 μs, N/A, 20	65	Achieved a fracture healing time of 43.2 ± 10.9 days in the ultrasound group and 62 ± 19.2 days in the control group.	N/A
[89]	51	Tibial shaft fractures	1.5 MHz, 30 mW/cm^2^, 1 kHz, 200 μs, N/A, 20	112	The group treated with LIPUS had significantly better bone healing after treatment than the control group.	N/A
[90]	30	Tibial fractures	1.5 MHz, 30 mW/cm^2^, 1 kHz, 200 μs, N/A, 20	90	LIPUS-treated group was found to have the least complications and showed statistically better healing.	N/A
[91]	30	Lateral malleolus fractures	1.5 MHz, 30 mW/cm^2^, 1 kHz, 200 μs, N/A, 20	42	N/A	No statistically significant effect of LIPUS on fracture healing of lateral malleolus was observed.
[92]	13	Delayed union of fibula after high tibial osteotomy	1.5 MHz, 30 mW/cm^2^, 1 kHz, 200 μs, N/A, 20	60–120	LIPUS treatment accelerated the clinical healing of delayed fibular union by increasing the osteoid thickness, mineral deposition rate, and bone volume in the nascent bone formation area.	LIPUS treatment did not alter osteoid thickness or the mineral apposition rate in cancellous bone regions.
[93]	27	Osteotomy sites after forearm bone shortening osteotomies	1.5 MHz, 30 mW/cm^2^, 1 kHz, 200 μs, N/A, 20	84	LIPUS treatment was found to reduce the cortical healing time by 27%; the healing time of endosteum was shortened by 18%.	N/A
[94]	21	Recovery after intraoral vertical ramus osteotomy	1.5 MHz, 30 mW/cm^2^, 1 kHz, 200 μs, N/A, 20	21	LIPUS-treated patients had increased bone mineral density and faster bone healing than patients in the control group.	N/A
[95]	13	Delayed union after fibular osteotomy	1.5 MHz, 30 mW/cm^2^, 1 kHz, 200 μs, N/A, 20	150	LIPUS treatment significantly increased the size of blood vessels. There was a significant correlation between vessel size and osteoid volume during delayed healing.	LIPUS treatment did not change the number of blood vessels.
[96]	29	Nonunion	1.5 MHz, 30 mW/cm^2^, 1 kHz, 200 μs, N/A, 20	154	LIPUS can be used to treat challenging, established nonunions.	N/A
[97]	36	Underwent distraction osteogenesis (2 cm)	1.5 MHz, 30 mW/cm^2^, 1 kHz, 200 μs, N/A, 20	165	LIPUS application during callus distraction served as a useful adjunct therapy for distraction osteogenesis.	N/A
[98]	21	Tibial distraction osteogenesis	1.5 MHz, 30 mW/cm^2^, 1 kHz, 200 μs, N/A, 20	N/A	LIPUS treatment accelerated callus maturation by 27%, increased the daily radiographic callus density by 33%, and reduced the fixation time by 95 days compared to the control group.	N/A
[99]	30	Distraction osteogenesis of the tibia	1.5 MHz, 30 mW/cm^2^, 1 kHz, 200 μs, N/A, 20	N/A	The healing indexes of the anterior and medial cortices were significantly improved in the LIPUS group, exhibiting a markedly faster healing rate than the control group.	No significant difference in the external fixation index was found between the LIPUS and control groups.
[100]	23	Lower limb bone stress injuries	1.5 MHz, 117 mW/cm^2^, 1 kHz, 200 ms, N/A, 20	28	N/A	No significant differences were found between the LIPUS treatment and placebo groups in changes to MRI grading and bone marrow edema size.
[101]	44	Chevron osteotomy	1.5 MHz, 30 mW/cm^2^, 1 kHz, 200 μs, N/A, 20	21	The LIPUS group showed an increase in the distal metatarsal articular angle compared to the placebo group.	The hallux valgus angle, intermetatarsal angle, sesamoid position, and metatarsal index showed no statistically significant differences between the LIPUS treatment and placebo groups.
[102]	142	Scaphoid nonunion	1.5 MHz, 30 mW/cm^2^, 1 kHz, 200 μs, N/A, 20	56	N/A	LIPUS therapy exerted no significant effect on healing time in patients with established scaphoid nonunions.
[103]	20	Postmenopause	1.5 MHz, 30 mW/cm^2^, 1 kHz, 200 μs, N/A, 20	180	N/A	LIPUS treatment showed no change in the overall or trabecular bone mineral density of the distal radius.

* F, frequency; I_SATA_, space-averaged time-averaged intensity; PRF, pulse repetition frequency; PD, pulse width; PDC, pulse duty cycle; T, exposure time; LIPUS, low-intensity pulsed ultrasound; N/A, not available or not applicable.

**Table 4:** Details of animal studies on the effect of LIPUS on bone remodeling disorders.

Ref.	Species	Model	Parameters (F, I_SATA_, PD, PRF, PDC, T) *	Positive Results	Negative Results
[59]	Rat	Ovariectomy	1.0 Hz, 30 mW/cm^2^, 1 kHz, 200 μs, N/A, 20	N/A	LIPUS treatment had no significant effect on bone loss in the distal femur or proximal tibia.
[114]	Rabbit	Leg fracture	N/A, 200 mW/cm^2^, N/A, N/A, N/A, 3	Fractures treated with ultrasound healed 35 days faster than those in the control group.	N/A
[115]	Rat	Femoral osteotomy Model	7.5 MHz, 11.8 mW/cm^2^, 1 Hz, 1 ms, N/A, 10	Ultrasound treatment enhanced bone mineral density in the fracture region and promoted the acceleration of fracture repair.	N/A
[116]	Rabbit	Transverse osteotomy of the tibial diaphysis	1.5 MHz, 30 mW/cm^2^, 1 kHz, 200 μs, N/A, 20	Radiographic assessment revealed a substantial maturity in bone formation within the LIPUS group, while the control group exhibited only immature bone regeneration.	N/A
[117]	Rabbit	Mid-tibia1 osteotomy	1.5 MHz, 30 mW/cm^2^, 1 kHz, 200 μs, N/A, 20	LIPUS treatment resulted in an enlarged distraction callus and decreased fibrous tissue in the tibia.	LIPUS treatment showed no significant effect on the mechanical properties and density of the regenerated bone.
[118]	Sheep	Midshaft osteotomy of the left tibia	1.0 MHz, 30 mW/cm^2^, 1 kHz, 200 μs, N/A, 20	The LIPUS treatment group demonstrated a 24-day reduction in healing time compared to the control group, and also exhibited enhanced cortical bone density and ultimate strength.	N/A
[119]	Canine	Tibial plateau leveling osteotomy	1.5 MHz, 30 mW/cm^2^, 1 kHz, N/A, 20%, 20	N/A	The LIPUS and sham groups showed no significant difference in radiographic bone healing or limb function via objective gait analysis.
[120]	Horse	Fracture gap of the fourth metacarpal bone	1.5 MHz, 44 mW/cm^2^, 1 kHz, 2 ms, 33%, 40	N/A	No significant differences were observed in any radiographic or histological parameters between the LIPUS treatment group and the control group.
[121]	Rat	Ovariectomy-induced osteoporotic fracture	1.5 MHz, 30 mW/cm^2^, 1 kHz, 200 μs, N/A, 20	LIPUS treatment accelerated fracture healing by enhancing callus formation, angiogenesis, and callus remodeling.	N/A
[122]	Mice	Fracture of femur	1.5 MHz, 30 mW/cm^2^, 1 kHz, 200 μs, N/A, 20	LIPUS treatment halved the endochondral bone remodeling period to 10 days and accelerated femoral fracture healing.	N/A
[123]	Rat	Ovariectomized	1.0 MHz, 1500 mW/cm^2^, 1 kHz, 200 μs, N/A, 20	LIPUS treatment mitigated estrogen deficiency-induced bone loss and fragility by enhancing bone formation.	N/A
[124]	Rat	Ovariectomy	1.0 MHz, 30 mW/cm^2^, 1 kHz, N/A, N/A, 20	LIPUS stimulation promoted an increase in femoral wet weight in ovariectomized rats.	N/A
[125]	Rat	Ovariectomy and osteoporotic bone defect	1.5 MHz, 30 and 150 mW/cm^2^, 1 kHz, 200 μs, N/A, 20	LIPUS at 150 mW/cm^2^ showed higher drill-hole healing after 3 weeks and greater improvements in bone density, microstructure, and biomechanical properties by 6 weeks compared to 30 mW/cm^2^.	N/A
[126]	Mice	Ovariectomy	1.5 MHz, 30 mW/cm^2^, 1 kHz, 200 μs, N/A, 20	LIPUS treatment significantly enhanced bone microarchitecture parameters compared to the control group, as evidenced by increased bone volume/tissue volume, trabecular number, trabecular pattern factor, and polar moment of inertia.	N/A
[18]	Rat	Ovariectomy	1.5 MHz, 5, 30 and 100 mW/cm^2^, 1 kHz, 200 μs, N/A, 20	LIPUS treatment improved bone microarchitecture, increasing the bone volume/total volume ratio, the apparent level elastic modulus, and the mechanical strength of trabeculae.	N/A

* F, frequency; I_SATA_, space-averaged time-averaged intensity; PRF, pulse repetition frequency; PD, pulse width; PDC, pulse duty cycle; T, exposure time; LIPUS, low-intensity pulsed ultrasound; N/A, not available or not applicable.

## Data Availability

Not applicable.
